# Childhood Neglect and Loneliness: The Unique Roles of Parental Figure and Child Sex

**DOI:** 10.3390/bs14060442

**Published:** 2024-05-24

**Authors:** Megan Ho, Julie Aitken Schermer

**Affiliations:** 1 Department of Psychology, University of Western Ontario, London, ON N6A 5C2, Canada; mho284@uwo.ca; 2 Department of Psychology and Management and Organizational Studies, University of Western Ontario, London, ON N6A 5C2, Canada

**Keywords:** childhood, neglect, emotions, loneliness

## Abstract

There is a well-supported link between experiences of childhood neglect and levels of loneliness in adulthood, with emotional neglect from caregivers being predictive of loneliness. However, current research has yet to explore additional, sex-linked factors that influence this relationship. This study investigates the impact of different neglect types on loneliness, with a focus on the parental figure involved and the child’s sex. It was hypothesized that men who experienced emotional neglect from their fathers would score higher in loneliness compared to other parent–child combinations. The findings showed no significant differences in father–son relationships within the context of emotional neglect. However, there was a significant difference in father–son relationships in the context of supervision neglect and loneliness outcomes, relative to all other parent combinations. Consistent with existing research, emotional neglect emerged as the strongest predictor of loneliness. Additionally, sex differences were observed, with women experiencing greater levels of loneliness stemming from neglect compared to men. These findings help address the knowledge gap present in childhood neglect research, with the goal of understanding the long-term consequences of adverse childhood experiences.

## 1. Introduction

The phrase ‘neglect of childhood neglect’ highlights the lack of existing research pertaining to childhood neglect despite its high prevalence in over one in five children [1]. The consequences of childhood neglect are well-documented, including future loneliness as an adult [2]. However, it is unclear whether neglect outcomes are affected by the specific caregiver (i.e., a mother or a father) who neglects the child. Mothers and fathers uniquely impact certain domains of children’s emotional development [3] and, conversely, it is possible that neglectful behaviours from a specific caregiver may impact feelings of loneliness more strongly. Research suggests that a child’s sex might also play a role within neglect outcomes, as certain aspects of men’s emotional processing are more significantly impacted by paternal figures than maternal figures [4,5,6]. The present study will focus on who neglected the child, as well as the child’s sex, as predictor variables of subsequent loneliness resulting from childhood neglect.

### 1.1. Childhood Neglect

There are varying definitions of childhood neglect; however, for this study, childhood neglect is defined as the failure to provide for a child in any aspect that can cause harm to their development [7]. Childhood neglect is a type of childhood maltreatment, which encompasses all types of treatment that can harm a child’s development or dignity [7]. Other types of maltreatment, in addition to neglect, include physical abuse, emotional abuse, and sexual abuse [8]. Defining neglect is a critical part of distinguishing its consequences relative to other forms of maltreatment, especially given that neglect is documented as the most frequent type of maltreatment, accounting for 60% of all proven child protective service cases [9].

Existing research on childhood neglect is not representative of its high prevalence rate. For example, research pertaining to the long-term health outcomes of childhood maltreatment assesses neglect in less than 25% of studies, despite it being reported approximately 60% of the time [10]. These statistics illustrate a dramatic misalignment between the need for childhood neglect research and the studies that are conducted. Furthermore, when neglect is studied, it is not examined as thoroughly as other types of maltreatment, which contributes to the limited research available on the topic. In particular, research rarely distinguishes between the subtypes of neglect, whereas the subtypes of abuse are examined as separate, independent variables [11,12]. This limited research also restricts the ability to conduct more comprehensive analyses on the issue of neglect, including meta-analyses and the identification of moderator variables [12]. The lack of attention towards childhood neglect in research is not new as the phrase “neglect of childhood neglect” has been referenced in research for over four decades [13]. As a result, there continues to be a lack of understanding of the factors contributing to childhood neglect and its outcomes.

The importance of childhood neglect is not only illustrated within its prevalence rate, but also in the severity of its consequences. Childhood neglect is associated with a wide variety of negative, short-term, and long-term outcomes, including disruptions in behaviour, cognition, and emotional development [9,14]. Specifically, neglected children are at an increased risk of having violent and anti-social behaviours, lower intelligence scores, experiencing greater negative affect, and developing personality disorders, relative to non-neglected children [13,14] These consequences are often underestimated in comparison to abuse; however, the long-term effects of neglect are comparable and in some cases are more severe [15,16,17]. For example, relative to abused children, neglected children are at higher risk of cognitive and academic deficits, social withdrawal, and cognitive functioning impairments [13,18]. The extensive impact of neglect is also illustrated in its effects at the neural level, as it is predicted to be the most detrimental form of maltreatment for brain development [19,20]. As reflected in the disproportionate research on this topic relative to abuse, the impact of neglect is underestimated, ultimately contributing to the deficiency in childhood neglect research. 

The pervasive social consequences associated with neglect are particularly harmful to development, as they serve as risk factors for other aversive outcomes. For example, childhood neglect is associated with the development of an anxious attachment style, characterized by an oversensitivity to rejection cues, and exaggerated, negative emotions in relationships [21,22,23]. These interpersonal reactions ultimately lead to lower-quality relationships and higher relational conflict, which increases the risk of anxiety, depression, lower quality of life, and more severe health outcomes [24,25,26,27]. Additionally, lower quality relationships serve as a significant risk factor for loneliness, a feeling that occurs when intimate and social needs are unmet, reflecting a discrepancy between one’s perceived and actual social relationships [28,29]. The development of loneliness, stemming from anxious attachment behaviours, illustrates the far-reaching consequences of neglect. 

### 1.2. Loneliness and Childhood Neglect

Loneliness is a prominent outcome of neglect as it is not just an indirect consequence, mediated by anxious attachment and lower quality relationships, but is also directly predicted by neglect experiences [30,31]. Thus, neglected individuals may be susceptible to more severe levels of loneliness as a result of encountering both direct and indirect risk factors [31,32,33]. The indirect and direct nature of this relationship highlights the strength of neglect as a predictor of loneliness, making it an ideal relationship for exploring additional factors that might influence the consequences of neglect. The consequences of loneliness are severe, including a range of physical, cognitive, and emotional outcomes, such as increased mortality, diminished cognitive control, increased risk of developing personality disorders, and increased rates of suicide [2,29,34], which highlights the need to better understand the additional factors that affect neglect and loneliness. 

The complex interplay between neglect and loneliness is also evident in their similar frameworks and function as a cycle that enhances isolation behaviours. Specifically, neglect follows the parental rejection feedback loop, which suggests that those who experience childhood rejection become hypervigilant and hypersensitive to rejection, and further isolate themselves to prevent further damage [35]. Similarly, loneliness functions in the same way, where individuals who are lonely tend to be more fearful, view the world as threatening, further isolate themselves, and become more fearful [29]. Given the comorbidity of neglect and loneliness, there is a strong inclination towards self-isolation, which further amplifies the risk for loneliness by contributing to lower quality relationships, and deficits in belonging needs [29,35,36] This relationship further illustrates the degree to which neglect and loneliness are interrelated, as their inherent behavioural responses can lead to even stronger feelings of loneliness. 

The risk and severity of loneliness varies based on the type of childhood maltreatment that is experienced. Broadly, childhood maltreatment is associated with enduring feelings of loneliness, even into young adulthood [37,38]. However, emotional neglect is particularly impactful and is associated with greater levels of loneliness compared to other types of neglect and maltreatment, including physical, verbal, and sexual abuse [2,31]. The influence of emotional neglect on loneliness outcomes can be explained in terms of the nature of these experiences, as emotional neglect is communicative of a personal, psychological sense of rejection, which, as highlighted in the parental rejection feedback loop, triggers isolation behaviours and subsequently increases loneliness risk [33,35]. Although the severity of loneliness differs based on neglect type, minimal research has explored additional factors that further exacerbate loneliness outcomes. 

### 1.3. The Role of Caregivers

The responsibility of child rearing has shifted to both parents, as opposed to previously being predominantly a mother’s responsibility [39]. Despite this shift, there remains a differential impact of maternal and paternal parenting on children’s development, specifically within domains pertaining to loneliness vulnerability. For example, relative to maternal parenting, paternal parenting plays a particularly significant role in children’s development of quality, platonic and romantic relationships [3,40,41]. This connection suggests that, conversely, neglect by a paternal figure has a greater impact in undermining future relationship quality, which heightens loneliness risk [30]. Furthermore, paternal parenting also plays a unique role in developing quality sibling relationships, which can help compensate for the lack of social support from other, lower-quality relationships [42]. Thus, the neglect from a paternal parent might diminish this protective factor against loneliness. According to attachment theory, it is unclear whether secure attachment to one parent more strongly influences overall development [43]. However, previous studies highlight the unique impact of paternal parenting in developing quality relationships, suggesting that paternal neglect can have a differential impact on loneliness outcomes. Overall, there remains insufficient research to determine whether the individual parent is a significant variable in the relationship between neglect and loneliness [31].

Existing research suggests that the impact of paternal parenting differs based on a child’s sex. Specifically, emotional maltreatment from a paternal figure impacts males to a greater extent than females within several domains related to loneliness. For example, paternal emotional manipulation negatively impacts boys’ self-esteem to a greater extent than girls’ self-esteem [4]. Self-esteem is negatively correlated with loneliness, and this relationship implies that males who are emotionally manipulated by their fathers are at a greater risk of being lonely [6]. Positive paternal parenting also impacts males differently, as a higher-quality, father–son relationship is related to lower emotional reactivity to stressful events, serving as a protective factor against loneliness [5,44,45]. The unique paternal impact on emotional reactivity was lacking in father–daughter pairs [5], which supports the theorized importance of a father’s parenting behaviours in men’s risk for loneliness development. Further evidence supporting the influence of paternal behaviours on outcomes is illustrated in Loos and Alexander (1997)’s study, where men’s loneliness is predicted by paternal emotional neglect but not maternal emotional neglect [37]. 

Although there is a strong theoretical connection between paternal maltreatment and men’s loneliness outcomes, minimal research has examined this relationship. Loos and Alexander (1997)’s work is the only identifiable study that investigates the interaction between parent and child within the context of neglect type and loneliness [37]. However, several important changes have occurred since the differential impact of parental neglect was investigated, such as greater gender equality as indicated in a reduction in the gender wage gap and an increase in women’s educational attainment and employment [46]. These patterns introduce the possibility that previous gender or sex differences and their respective impacts may have decreased. Another critical change includes the occurrence of the COVID-19 pandemic, where mental health discussions became less stigmatized [47], and may have influenced the salience of loneliness. These important societal changes suggest that previous relationships between the perpetrator of childhood neglect and loneliness may have changed as well. Overall, the knowledge of additional factors affecting childhood neglect outcomes are scarce, ultimately preventing a more comprehensive understanding of neglect [13]. The lack of knowledge in these domains reaffirms the ‘neglect of childhood neglect’ that continues to resurface within childhood maltreatment research. 

### 1.4. Present Study

The present study aimed to address whether the relationship between the perpetrator of childhood neglect and the child influences loneliness outcomes. This study also examined the influence of neglect type, the sex of the neglected individual, and the parental figure on loneliness outcomes. To better understand the long-term effects of childhood neglect, this study surveyed young adults, where participants reported on their neglect experiences, retrospectively. Measures of previous childhood neglect experiences, the parental figure of the neglect, and current levels of loneliness were collected using self-report measures. 

There were two hypotheses for the current study. It was hypothesized that those who experienced higher levels of emotional neglect would score higher on levels of loneliness compared to the other types of neglect, which is consistent with previous neglect and loneliness research [2,31]. It was also hypothesized that men who were emotionally neglected by their fathers would score higher in loneliness compared to all other combinations of child–parent relationships. This prediction aligns with previous research on the significance of the father–son relationship [37]. This study ultimately serves to minimize the knowledge gap present in childhood neglect research and inform strategies designed to decrease the development of loneliness stemming from adverse childhood experiences. 

## 2. Materials and Methods

### 2.1. Participants

This study consisted of a total of 606 participants (370 men and 236 women)—above the minimum sample size of 250 participants, which represents the number of responses required for correlations to stabilize [48]. The age of participants ranged from 17 to 37 years old, and the average age was 18.33 years (*SD* = 1.41). Participants were recruited from a first-year business management program. All participants completed an informed consent form and were required to read English to participate in this study to ensure their accurate understanding of the survey items. According to the Tri-Council Policy Statement on research conducted with humans in Canada, university students do not require parental consent to participate in research, thus participants who were 17 years old did not require additional consent from parents or guardians. Participants received compensation in the form of a research credit regardless of completing the study or not. Ethics approval was obtained from the university’s Non-Medical Research Ethics Board (Protocol #124058). 

### 2.2. Procedure

Participants accessed the Qualtrics survey through SONA, an on-line platform used for participant recruitment, study administration, and compensation in research. Participants completed the survey at their leisure, using their own devices. Completion required about 20 min. Participants completed an attention check question and were asked whether they had answered honestly and if their responses could be used. Those participants who indicated that they did not answer honestly were excluded from the analysis. There were 638 participants that enrolled in the study and data from 32 participants were excluded based on this criterion. At the conclusion of the study, participants were given a debriefing form that contained additional resources to consult if the study triggered emotional upset, as well as references to relevant research articles.

### 2.3. Measures

#### 2.3.1. Mother Childhood Neglect Scale, Short Form (MCNS-SF)

Childhood neglect experiences were measured using a revised version of the Mother Childhood Neglect Scale (MCNS-SF) [49]. The MCNS-SF was originally designed as a self-report measure for caregivers to report neglectful behaviour, and for the purposes of this study, the subject of the sentence was changed to “my parent/caregiver” instead of “I” to measure participants’ recall of parental neglect. The revised version of the MCNS-SF can be found in Appendix A. The MCNS-SF was used because it is a reliable and valid measure [49]. The MCNS-SF consists of eight items that measure the four subscales of neglect: emotional neglect, physical neglect, cognitive neglect, and supervision neglect. Each subscale consists of two items. Emotional neglect items measured a lack of affection and support, physical neglect items measured a deficit of items such as food and clothing, cognitive neglect measured a lack of mental stimulation, and supervision neglect measured setting boundaries and correcting misbehavior [49]. In the original MCNS-SF, the items were scored on a 4-point Likert scale for each item, ranging from strongly agree to strongly disagree. However, to reduce ambiguity in the responses, the version of the MCNS-SF in this study consisted of true or false response options. Responses that indicated ‘true’ were coded with a score of 1, and responses that indicated ‘false’ were coded with a score of 2. Thus, the total scores of the MCNS-SF in this study ranged from 8 to 16, and higher scores indicated greater experiences of neglect. In the present study, coefficient alpha estimates for the two true/false item scales were 0.638 for the emotional neglect items, 0.375 for the supervision neglect items, 0.263 for the physical neglect items, and 0.434 for the cognitive neglect items.

#### 2.3.2. Open-Ended Question/Response

At the end of the MCNS-SF, participants were asked, “For the questions you just completed, which parent were you thinking about when you answered?”. These responses were ‘dummy coded’ to include the categorical predictor of parent sex in the quantitative analysis. Mother was coded as ‘1′, father was coded as ‘2′, both parents were coded as ‘3′, and other was coded as 4. 

#### 2.3.3. Three-Item UCLA Loneliness Scale 

Self-report loneliness was measured using the Three-Item UCLA Loneliness scale [50]. The reliability and validity of this scale is comparable to the Revised UCLA Loneliness Scale (R-UCLA), which is one of the standard loneliness measures used in research [50]. Each of the three items in the scale was assessed using a 3-point Likert scale, consisting of the responses ‘hardly ever’, ‘some of the time’, and ‘often’. The sum of all three responses yielded a total score on the loneliness scale that ranged from 3 to 9. Higher scores indicated greater loneliness experiences. In the present study, coefficient alpha for the three-item loneliness scale was 0.714.

#### 2.3.4. Demographic Questionnaire

Participants completed a demographic questionnaire that surveyed their age, assigned sex at birth, and gender. Participants’ age was assessed using an open-ended question. Participants’ sex was assessed using a multiple-choice question that inquired about participants’ sex at birth, and the responses options consisted of ‘male’ and ‘female’. Participant gender was assessed using an open-ended question that asked, “what do you define as your gender?”

### 2.4. Analysis

The first a priori hypothesis, that emotional neglect is a stronger predictor of loneliness compared to the other three types of neglect, was tested using a regression analysis to examine the significance of each neglect type in predicting loneliness scores. The criterion for significance was a standard two-tailed test, with an alpha level of 0.05. All assumptions of the regression analysis were met, except for the assumption of normality. Additional transformations of the data were not performed as both correlations and regression analyses are robust even when normality assumptions are not met. Participant data were excluded using listwise deletion to ensure that all the variables examined in the analysis were present. 

The second a priori hypothesis examines the loneliness of emotionally neglected males and females based on their specific caregiver. It was hypothesized that males who were emotionally neglected by their fathers would score higher in loneliness, compared to all other specific parent–child combinations. This hypothesis was tested by computing the correlations between emotional neglect scores and loneliness scores for men neglected by their fathers, and for all other parent–child relationships. The correlations of the other three parent–child relationships (women neglected by their mothers, women neglected by their fathers, and men neglected by their mothers) were grouped together due to the uneven number of participants within each category, with only eight females indicating neglect experiences from their father. A Fisher’s *r*-to-*z* transformation was conducted to convert the two groups of correlations into *z*-scores, and the correlations were directly compared using a *z*-test.

Five additional analyses were conducted to explore relationships between neglect type, participant’s sex, parental figure, and loneliness. The first analysis compared the relationship between neglect and loneliness for men neglected by their fathers in the context of physical, cognitive, and supervision neglect. The second examined men emotionally neglected by their fathers versus men emotionally neglected by their mothers. The third analysis compared the relationship between the other three types of neglect and loneliness, between father–son relationships and mother–son relationships. The fourth analysis compared the relationship of neglect and loneliness between men and women to examine the influence of participant’s sex. The fifth analysis examined the differences in neglect and loneliness based on the parental figure of neglect. Each analysis involved computing the correlations of a specific neglect measure and loneliness for each participant–parent combination, conducting Fisher’s *r*-to-*z* transformations to convert the correlations into *z*-scores, followed by *z*-tests to compare the correlations. Pairwise deletion was used to filter the data and the significance level was set at the standard level of *p* < 0.05. 

## 3. Results

### 3.1. Descriptives

Of the participants who reported experiencing neglect, 287 participants reported neglect experiences from their mother, 52 participants reported neglect experiences from their father, and 208 participants reported neglect experiences from both parents. Furthermore, upon examining the sex of participants who reported experiencing neglect, 158 men reported neglect experiences from their mother, 44 men reported neglect experiences from their father, and 133 men reported neglect experiences from both parents. For women, 129 reported neglect experiences from their mother, eight reported being neglected by their father, and 75 reported neglect experiences from both parents. Table 1 reports the mean and standard deviation values for the neglect scales for each participant sex by parent combination.

### 3.2. Correlations

The correlations between the neglect scales and loneliness can be found in Table 2. There is a small, significant correlation between loneliness and emotional neglect, as well as loneliness and cognitive neglect. There is also a small, significant correlation between emotional neglect and physical neglect and a strong, significant correlation between emotional neglect and cognitive neglect. There is a weak, significant correlation between cognitive neglect and supervision neglect, as well as a moderate, significant correlation between cognitive neglect and physical neglect. There is also a weak, significant correlation between supervision neglect and physical neglect.

### 3.3. Hypothesis 1

A multiple linear regression was performed to assess the effect of the four types of neglect in predicting loneliness scores. The results of the regression are shown in Table 3 and reveal that for the overall model, neglect is a significant predictor of loneliness, *R*^2^ = 0.05, *R*^2^ adjusted = 0.04, *F*(4, 583) = 6.73, *p* = < 0.001. The four dimensions of neglect together account for 5% of the variance in loneliness. Emotional neglect was the only subtype of neglect that was a significant predictor of loneliness, whereas supervision, physical, and cognitive neglect were not significant predictors of loneliness. The missing data were random, and the model was based on complete responses from 588 individuals following listwise deletion.

### 3.4. Hypothesis 2

A bivariate correlational analysis was conducted between emotional neglect scores and loneliness scores for men neglected by their fathers, *r*(44) = 0.235, *p* = 0.125, and other parent–child relationships in the context of emotional neglect, *r*(548) = 0.189, *p* < 0.001. Fisher’s transformation test yielded a *z*-statistic of 0.297 and was not significant, *p* = 0.38. 

### 3.5. Exploratory Analyses

The first exploratory analysis compared men neglected by their fathers to other parent–child relationships, based on the other three types of neglect and loneliness. The correlations between physical neglect and loneliness for men neglected by their fathers, *r*(44) = −0.198, *p* = 0.197, and other parent child relationships, *r*(550) = 0.04, *p* = 0.39, were compared, and the Fisher’s *r*-to-*z* transformation test was not significant, with a *z*-statistic of −1.21, *p* = 0.11. The correlations for cognitive neglect and loneliness were computed for men neglected by their fathers, *r*(44) = 0.130, *p* = 0.40, and other parent–child relationships, *r*(549) = 0.173, *p* <0.001. The results of the Fisher’s *r*-to-*z* transformation test yielded a *z*-statistic of −0.25 and was not significant, *p* = 0.40. This procedure was repeated for supervision neglect for men neglected by their fathers, *r*(44) = −0.275, *p* = 0.07 and other parent–child relationships, *r*(550) = −0.01, *p* = 0.91. The results of the Fisher’s *r*-to-*z* transformation test yielded a *z*-statistic of −1.71 and was significant, *p* = 0.04. 

The second exploratory analysis examined the relationships between emotional neglect and loneliness for men neglected by their father, compared to men neglected by their mother. Upon conducting the independent samples *t*-test, the results of Levene’s test for equal variances revealed significant differences between the groups, *F* = (1, 25.09), *p* < 0.001. The values were adjusted accordingly, and the results reveal that there is a significant difference in experienced emotional neglect between men neglected by their fathers, compared to their mothers, *t*(60) = −2.31, *p* < 0.001. A bivariate correlation analyses and Fisher’s *r*-to-*z* transformation tests were then used to compare the correlations of emotional neglect and loneliness in men that reported experiencing neglect from a single parental figure. The correlations between men emotionally neglected by their fathers, *r*(44) = 0.235, *p* = 0.13 were compared to men emotionally neglected by their mothers, *r*(155) = 0.088, *p* = 0.28. Fisher’s *r*-to-*z* transformation test yielded a *z*-statistic of 0.86, and was not significant, *p* = 0.20. 

The third exploratory analysis separately compared the correlations between loneliness and the other three types of neglect, between men neglected by their fathers and men neglected by their mothers. The results of the exploratory analysis were not significant. Specifically for physical neglect, the correlations between men neglected by their fathers, *r*(44) = −0.198, *p* = 0.20, and men neglected by their mothers, *r*(156) = −0.04, *p* = 0.66, yielded a *z*-statistic of −0.91, *p* = 0.18. For cognitive neglect, the correlations between men neglected by their fathers, *r*(44) = 0.130, *p* = 0.40, and men neglected by their mothers, *r*(156) = 0.09, *p* = 0.29, yielded a *z*-statistic of 0.23, *p* = 0.41. For supervision neglect, the correlations between men neglected by their fathers, *r*(44) = −0.28, *p* = 0.13, and men neglected by their mothers, *r*(156) = −0.04, *p* = 0.58, yielded a *z*-statistic of 0.97, *p* = 0.17.

The fourth exploratory analysis investigated the sex differences in the relationship between neglect and loneliness. The bivariate correlational analysis between total neglect and loneliness was significant for women, *r*(224) = 0.238, *p* <0.001, but not for men, *r*(363) = 0.087, *p* = 0.10. The correlations were also compared using Fisher’s *z*-transformation test, and the results were significant, with a *z*-statistic of 1.82, *p* = 0.03. 

The fifth exploratory analysis examined the role of the specific parental figure on loneliness outcomes. Levene’s test for equality of variances was not significant, *F* (1, 334) = 0.01, *p* = 0.74. The results of the *t*-test revealed that the parental figure was not significant in predicting loneliness, *t*(546) = −0.143, *p* = 0.89. 

## 4. Discussion

The present study and the study by Loos and Alexander [37] appear to be the only available studies investigating the interaction between parental figure and sex within the context of neglect and loneliness. The present study extends the findings of Loos and Alexander [37] by examining the influence of parent–child relationships on loneliness, beyond emotional neglect. Whereas current findings focus on emotional neglect and loneliness outcomes within specific parent–child relationships, the present study is the first to examine the differences across parent–child relationships, pertaining to several neglect types and loneliness.

### 4.1. Neglect Subtypes as Predictors of Loneliness

As hypothesized, emotional neglect is the strongest predictor of loneliness. This finding is consistent with existing research suggesting that children who experience emotional neglect tend to be lonelier later in life, compared to those who experience other forms of maltreatment [2,31,37,38]. Existing research is predominantly focused on the outcomes of emotional and physical neglect [31] and this study was the first to examine supervision and cognitive neglect as independent predictors of loneliness. Although supervision and cognitive neglect were not significant predictors of loneliness, the positive, moderate correlation between cognitive and emotional neglect suggests that these constructs may be interrelated. Future research should investigate the potential relationship between emotional and cognitive neglect, as well as the risk of cognitive neglect in predicting other long-term, aversive outcomes. 

Although outside of the scope of this study, a potential explanation behind the lack of significance of supervision and cognitive neglect can be inferred from the neglect items. Specifically, these items measure care in educational and intellectual domains as opposed to emotional support [49], which relative to emotional neglect, might not convey strong feelings of psychological rejection when they are not met. Consequently, these types of neglect might not serve as risk factors to loneliness as they do not trigger the isolation responses tied to parental rejection [33,35]. Minimal research examines these neglect subtypes, and their relationship to loneliness warrants further investigation. Overall, the significance of the overall regression model in this sample demographic reinforces the extensive impact of neglect, with loneliness enduring into early adulthood [13,18]. 

Contrary to previous research, physical neglect was not a significant predictor of loneliness [31,51,52,53]. These results may be attributed to the narrow range of responses inherent within a dichotomous scale, which limits response variability by capturing more extreme instances of neglect [54]. Furthermore, as reflected in Western University’s Equity Census [55], the sample consisted of university students, predominantly from wealthier backgrounds. Physical neglect is less common in higher income families [56] and the prevalence of physical neglect is likely diminished within this group. Thus, the impact of using a dichotomous scale, as well as a homogenous sample, may have influenced responses to physical neglect items. Future research should ensure the use of measures that consider a range of neglect experiences and be aware of how neglect experiences might vary within different populations. The use of a more representative sample is also critical to ensure that results can be extended beyond the sample demographic. 

### 4.2. Emotional Neglect and Loneliness for Men Neglected by Fathers 

The relationship between emotional neglect and loneliness is consistent across all parent–child relationships. Thus, the findings do not support the hypothesis for a unique relationship between loneliness and emotional neglect for men neglected by their fathers, relative to all other individuals who were emotionally neglected by other parental figures. Further analysis showed that although men tend to experience greater levels of emotional neglect from fathers as opposed to mothers, loneliness outcomes remained the same between groups. These findings extend the current understanding of parent–child relationships, suggesting that although previous research suggests that men’s loneliness is predicted by emotional neglect specifically by father figures [37], the relationship between emotional neglect and loneliness does not vary significantly across parent–child relationships. There remains insufficient research to reliably determine whether a paternal figure can influence neglect outcomes [31], thus additional research is needed to clarify the nature of this relationship. 

It is important to note that the survey items did not account for whether participants grew up in a single-parent household, but only asked participants to indicate which parent was responsible for the neglectful behaviours. Household composition may have served as a confounding variable, as loneliness outcomes might differ within single- and dual-parent households. Specifically, there is a higher risk for loneliness in single-parent households [57,58] and, in contrast, the presence of a second, attentive parent in dual-parent households might alleviate the effects of neglect. Furthermore, dual parents often divide responsibilities in childcare, which might influence whether their inattention in specific domains is perceived as neglect [59]. Single parents might not have this option of shared responsibility, which can also bias perceptions of neglect. Subsequent research should explore how family dynamics and family composition might affect neglect and loneliness outcomes. 

### 4.3. Other Neglect Types and Loneliness across Parent-Child Relationships

Physical and cognitive neglect outcomes were consistent within all parent–child relationships. No difference was hypothesized in these neglect domains; however, considering the potential loneliness confounding factor of single- and dual-parent households, further studies are needed to verify the reliability of these findings. 

### 4.4. Supervisory Neglect and Loneliness in Men Neglected by Fathers

There is a unique relationship between supervisory neglect and loneliness for men neglected by their father figures, relative to supervisory neglect in other parent–child relationships. Specifically, this relationship negatively correlated for father–son dyads, which runs contrary to previous research illustrating that supervisory neglect is positively associated with loneliness outcomes [60]. This inconsistency suggests that there is a unique interaction between sex and parental figure within supervisory neglect and loneliness; however, the mechanisms behind this interaction remain unclear. A potential rationale is that men behave differently in response to a lack of supervision, relative to women [61,62], and these sex differences might also extend to their emotional responses as well. Notably, subsequent analyses suggest that parental figure alone does not impact this relationship as there is no difference between neglect and loneliness in men who experienced supervisory neglect by their father or mother. These findings ultimately prompt further investigation into the developmental impacts associated with limited parental oversight, while considering the specific parental figure, as well as the child’s sex. 

### 4.5. Sex Differences in Neglect and Loneliness 

The present study provides evidence that suggests a difference in how women process neglect experiences. Specifically, women reported significantly greater levels of loneliness, compared to men, despite experiencing relatively similar levels of neglect. These findings contrast to earlier research, which indicates that there are no sex differences in either the effects of neglect or the experience of loneliness [31,63,64]. This discrepancy is difficult to explain conceptually, as the majority of research collectively examines the types of maltreatment, rather than isolating neglect [11,12]. The restricted sample demographic may have influenced these results as entering university is initially met with experiences of higher loneliness [64]. However, the sex differences in loneliness remain unaccounted for, which prompts further investigation into the interaction between sex, neglect, and loneliness. It is critical that future research explores how men and women might react differently to neglect to better understand the risks associated with aversive childhood experiences. 

### 4.6. Limitations and Future Directions

The limitations of this study mainly pertain to the specific measures. The items within the MCNS-SF prompt further investigation as the wording of several items appear vague, relative to others. For example, one of the items measuring emotional neglect enquires whether one’s parents ‘helped when I had problems’; however, this item fails to specify the nature of these problems. Additionally, given the time period in which this scale was developed, it is worth investigating whether these questions accurately measure neglect within their respective domains. In particular, an item measuring cognitive neglect enquires whether one’s parents ‘helped them with their homework’. A ‘false’ response implies neglect; however, the wording of this question fails to consider alternative scenarios, including parents opting to hire a tutor for their child due to time constraints or their own unfamiliarity with the topic. It would be helpful for future research to evaluate perceptions of these neglect items to ensure that the items are valid in measuring their respective constructs.

Although this research focused on neglect, its role as the sole predictor variable might have constrained the findings of this study. For example, other forms of maltreatment, including emotional abuse, are strong, unique predictors of loneliness [31,65]. Thus, unique relationships between a parental figure and a child’s sex may have been identified in the context of other maltreatment types. Subsequent studies should further explore the effects of parent–child relationships across various forms of maltreatment. 

Another limitation pertains to the survey item that enquired about which parental figure was viewed as neglectful, which may have insufficiently captured this information. This item asked participants which parental figures they thought of after completing all eight neglect items and in the case that they selected both parents, they were unable to specify which behaviours each parental figure was responsible for. Consequently, it is unclear whether children experienced all four types of neglect from both parents or specific types of neglect from one parent, which restricted the exploration of neglect and loneliness from a specific parent within the context of dual-parent households. Upon enquiring about the parental figure of neglect, careful consideration should be given to the way that questions are framed and how this might impact subsequent analyses. 

Furthermore, subsequent analyses were not conducted according to age, as most participants fell within the narrow range of 17–20 years old. However, it remains unclear whether loneliness, stemming from neglect, is robust across different age groups [31]. Other research examining various adverse childhood events reveals that levels of loneliness differ based on one’s current age and sex [66], suggesting that childhood neglect might follow a similar pattern. Future research should aim to clarify the relationship between neglect and loneliness across different age groups to gain a more comprehensive understanding of its effects across the lifespan. 

It is also important to acknowledge that the study did not consider additional factors such as participant gender and cultural background. Although minimal research has evaluated the difference between the effects of gender and sex within the context of neglect, the consideration of gender identity is important for fostering an inclusive and diverse understanding within research. Cultural demographics were not collected as the relationships between parental figure, sex, neglect type and loneliness were the key variables of interest in this study. However, there are cross-cultural definitions of neglect [67] and it is unclear how cultural variations in the definition of ‘neglect’ might influence loneliness outcomes. Given that the majority of neglect research is conducted within North America [12], a greater focus on the perceptions of neglect within different cultures is imperative to capture the complexity and diversity of child-rearing behaviours.

It is also important to note that causal inferences cannot be made based on the correlational nature of this study and future research is needed to clarify the reliability and validity of these findings. Nevertheless, this study provides important insights into the additional factors that contribute to neglect and loneliness.

There are several practical implications of the current findings. Psychological interventions, which are effective at reducing loneliness for both women and men [68], are applicable to those who are neglected, regardless of their sex. It is critical that these interventions adopt a lens of trauma-informed care, helping to promote resilience in moving forward, and ultimately reducing the persistence of loneliness [69]. Additionally, educators can assist in reducing loneliness risk by fostering more inclusive classroom environments to promote meaningful student interactions. Through increased vigilance towards instances of neglect, educators can direct their attention towards children who might be experiencing neglect and provide additional support. Given their role, educators can also help encourage and promote parental involvement within educational domains, ultimately helping to reduce neglectful behaviours. Childhood neglect research is imperative for informing successful interventions and future developmental research should aim to adopt a greater focus on neglect outcomes.

## Figures and Tables

**Table 1:** Neglect Descriptive Statistics for Parent–Participant Combinations.

Participant’s Sex	Parent	Emotional Mean (*SD*)	Supervision Mean (*SD*)	Physical Mean (*SD*)	Cognitive Mean (*SD*)
Men	Mother	2.16 (0.45)	2.94 (0.32)	2.05 (0.25)	2.25 (0.50)
	Father	2.43 (0.73)	2.86 (0.41)	2.11 (0.39)	2.48 (0.73)
	Both	2.12 (0.44)	2.92 (0.34)	2.04 (0.21)	2.20 (0.47)
Women	Mother	2.25 (0.55)	2.98 (0.32)	2.06 (0.24)	2.32 (0.55)
	Father	2.50 (0.76)	3.12 (0.35)	2.25 (0.46)	2.12 (0.35)
	Both	2.23 (0.56)	2.88 (0.37)	2.01 (0.11)	2.27 (0.55)

**Table 2:** Correlations Between Neglect Subtypes and Loneliness.

Variable	*n*	*M*	*SD*	1	2	3	4	5
1. Emotional Neglect	596	2.21	0.53	—				
2. Supervision Neglect	595	2.92	0.36	0.07	—			
3. Physical Neglect	595	2.06	0.26	0.22 **	0.16 **	—		
4. Cognitive Neglect	594	2.28	0.55	0.62 **	0.10 *	0.33 **	—	
5. Loneliness	592	5.73	1.58	0.19 **	−0.03	0.01	0.17 **	—

Note: * *p* < 0.05; ** *p* < 0.001.

**Table 3:** Predicting Loneliness Scores Based on Type of Childhood Neglect.

	Unstandardized Coefficients		Standardized Coefficients	*t*	Sig.
Model	B	Std. Error	Beta		
Constant	14.307	0.700		20.438	<0.001 **
Emotional	0.436	0.155	0.146	2.823	0.005 *
Supervision	−0.186	0.180	−0.042	−1.034	0.301
Physical	−0.308	0.270	−0.049	−1.141	0.254
Cognitive	0.280	0.154	0.097	1.815	0.070

Note. * *p* < 0.01, two-tailed; ** *p* < *0*.001, two-tailed.

## Data Availability

Data are available per reasonable request from the corresponding author.

## Appendix A

**Table A1:** Revised Survey Items of Mother Childhood Neglect Scale—Short form (MCNS-SF).

Question	True	False
My parent/caregiver kept me clean. *(P)*	1	2
My parent/caregiver made sure I went to school. *(S)*	1	2
My parent/caregiver did not care if I got into trouble at school. * *(S)*	1	2
My parent/caregiver gave me enough clothes to keep me warm. *(P)*	1	2
My parent/caregiver helped me when I had problems. *(E)*	1	2
My parent/caregiver helped me with homework. *(C)*	1	2
My parent/caregiver comforted me when I was upset. *(E)*	1	2
My parent/caregiver helped me to do my best. *(C)*	1	2

Note: Total neglect score is the sum of all eight items; * = reverse scored; *P* = measure of physical neglect; *S* = measure of supervisory neglect; *E* = measure of emotional neglect; *C* = measure of cognitive neglect.
